# Multiparameter flow cytometric detection and analysis of rare cells in *in vivo* models of cancer metastasis

**DOI:** 10.1093/biomethods/bpae026

**Published:** 2024-04-27

**Authors:** Mikaela M Mallin, Louis T A Rolle, Kenneth J Pienta, Sarah R Amend

**Affiliations:** Cancer Ecology Center, James Buchanan Brady Urological Institute, Johns Hopkins Medical Institute, Baltimore, MD, United States; Cellular and Molecular Medicine Graduate Training Program, Johns Hopkins School of Medicine, Baltimore, MD, United States; Cancer Ecology Center, James Buchanan Brady Urological Institute, Johns Hopkins Medical Institute, Baltimore, MD, United States; Cancer Ecology Center, James Buchanan Brady Urological Institute, Johns Hopkins Medical Institute, Baltimore, MD, United States; Cancer Ecology Center, James Buchanan Brady Urological Institute, Johns Hopkins Medical Institute, Baltimore, MD, United States

**Keywords:** circulating tumor cell (CTC), disseminated tumor cell (DTC), rare cell detection, flow cytometry (methods), metastasis (cancer metastasis)

## Abstract

Rapid and reliable circulating tumor cell (CTC) and disseminated tumor cell (DTC) detection are critical for rigorous evaluation of *in vivo* metastasis models. Clinical data show that each step of the metastatic cascade presents increasing barriers to success, limiting the number of successful metastatic cells to fewer than 1 in 1,500,000,000. As such, it is critical for scientists to employ approaches that allow for the evaluation of metastatic competency at each step of the cascade. Here, we present a flow cytometry-based method that enables swift and simultaneous comparison of both CTCs and DTCs from single animals, enabling evaluation of multiple metastatic steps within a single model system. We present the necessary gating strategy and optimized sample preparation conditions necessary to capture CTCs and DTCs using this approach. We also provide proof-of-concept experiments emphasizing the appropriate limits of detection of these conditions. Most importantly, we successfully recover CTCs and DTCs from murine blood and bone marrow. In Supplemental materials, we expand the applicability of our method to lung tissue and exemplify a potential multi-plexing strategy to further characterize recovered CTCs and DTCs. This approach to multiparameter flow cytometric detection and analysis of rare cells in *in vivo* models of metastasis is reproducible, high throughput, broadly applicable, and highly adaptable to a wide range of scientific inquiries. Most notably, it simplifies the recovery and analysis of CTCs and DTCs from the same animal, allowing for a rapid first look at the comparative metastatic competency of various model systems throughout multiple steps of the metastatic cascade.

## Introduction

There are currently upwards of 620,000 people living with metastatic breast, prostate, lung, colorectal, bladder, or melanoma cancer in the USA alone. This number is expected to increase to nearly 700,000 by 2025 [1]. A clinical diagnosis of metastasis requires the patient’s cancer cells to have completed the well-described metastatic cascade, consisting of five distinct steps: invasion by motile cells in the primary tumor site, intravasation into vasculature, survival within the circulatory system as circulating tumor cells (CTCs), extravasation into a distant secondary site as disseminated tumor cells (DTCs), and eventual colonization into a clinically detectable macrometastatic lesion [2]. While successful completion of the entire metastatic cascade appears common at the patient level, complete metastatic competency is a rare event at the cellular level. Barriers to metastasis compound along every step of the cascade, limiting a cell’s metastatic success [3]. Calculations using clinical data (i.e. frequencies of detectable CTCs, DTCs, and outgrown metastatic lesions in patients) indicate that the likelihood of any given CTC to successfully produce a metastatic tumor is less than 1 in nearly 1,500,000,000 [4]. A more nuanced understanding of where and when cancer cells that have initiated the metastatic process fail (or much more rarely, succeed) is crucial for the development of metastasis-intervention therapies. As such, it is critical for scientists to employ research approaches that discern which step(s) of the cascade present metastatic barriers to their model system of choice [5]. A rapid and robust method for the simultaneous evaluation of multiple steps of the cascade within one model system (i.e. simultaneous detection of CTCs and DTCs from a single animal), would (i) provide key insights into the metastatic potential of cancer cells in established *in vivo* metastasis models and (ii) aid in the development of metastasis-intervention strategies.

Here, we present an optimized, flow cytometry-based CTC and DTC detection approach for the identification of rare cells in murine tissues. Our approach leverages use of cancer cells that constitutively express cytosolic green fluorescent protein (GFP). GFP+ cells are introduced to mice using a variety of common injection techniques. At experimental endpoint, metastasis-relevant tissues (such as blood, bone marrow, or lung) are collected and processed for same-day flow cytometry analysis, using the presence of GFP signal as the basis for identification of CTCs and DTCs. Specifically, we describe the successful identification of blood CTCs and bone marrow DTCs recovered 6 weeks after subcutaneous injection of PC3-GFP-Luc human prostate cancer cells into 10-week-old, male, immunocompromised mice. We also demonstrate the widespread applicability of this approach, showing the subsequent successful identification of DTCs in the lungs of tail vein-injected mice. Furthermore, we illustrate the adaptability of this approach, offering an example of the additional fluorescence-based characterizations that can be multiplexed into the experimental design to further define and describe recovered CTCs and DTCs. For example: interest in the metastatic potential of polyploid cells has recently risen in the cancer field. A simple and convenient method to analyze the ploidy of metastasizing cells would expedite high-ploidy metastasis research. Accordingly, we report successful proof-of-concept ploidy analysis of known normal-ploidy (2N) and high-ploidy (4N+) CTCs and DTCs. Though this approach does not rely on any specific technological advancements, it is (to our knowledge) the first to combine cancer cell labeling with flow cytometry and ploidy analysis for the purpose of rare cell identification and characterization. We promote use of this approach for simple and rapid first looks at the comparative metastatic competency of various model systems.

## Materials and methods

**Figure 1.**
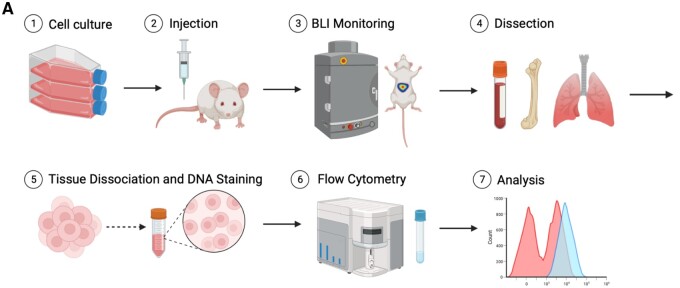
Workflow schematic. (A) Graphical abstract outlining the general steps of our featured technique, including cell culture, injection of cultured cells, monitoring of injected animals, metastasis-relevant tissue harvesting, tissue processing, flow cytometry-based data collection, and data analysis.

Broadly, this approach consists of seven steps: Cell culture, injection of cultured cells, monitoring of injected animals, tissue collection, tissue processing for flow cytometry, flow cytometry data collection, and data analysis (Fig. 1). Choice of cultured cell line, strain of mouse, injection route, monitoring method, and tissue to be collected are experiment specific and easily modifiable. Here, we present specific protocols for (i) culture of normal-ploidy or high-ploidy PC3-GFP-Luc prostate cancer cells, (ii) injection via subcutaneous or tail vein route, and (iii) collection and processing of blood and bone marrow (subcutaneous injections), or lung tissues (tail vein injections). Due to the live-cell nature of the flow cytometry, the tissue collection, tissue processing, and data collection must occur on the same day. A summary of the materials and equipment used is detailed in Table 1. Note that troubleshooting guidelines are detailed in Table 2, found in the Discussion section.

**Table 1.** Required materials and equipment

Application		Item	Vendor, Catalog Number
Cell culture	Normal Ploidy	PC3-GFP-Luc Cell Line^a^	–
		RPMI Medium 1640 + L-Glutamine 1X^a^	Gibco, 11875-119
		Qualified Fetal Bovine Serum^a^	Gibco, 26140-079
		Penicillin-Streptomycin^a^	Gibco, 15140-122
		Phosphate Buffered Saline pH 7.4 1X^a^	Gibco, 10010-049
		TyplE Express^a^	Gibco, 12604-013
		Trypan Blue Stain 0.4%^a^^,b^	Invitrogen, T10282
		Hemacytometer^a^^,b^	Hausser Scientific
		50 ml PP Conical Tube^a^	Falcon, 352098
		Sterile Biopur Eppendorf Tubes 5.0 ml^b^	Eppendorf, 0030119479
		Allegra X-30R Centrifuge^a^	Beckman Coulter
	High-Ploidy	Cisplatin	Adipogen Life Sciences, AG-CR1-3590-M250
*In vivo* injection	SC	Matrigel Matrix Basement Membrane	Corning, 356234
		29G 0.3 ml U-100 Insulin Syringe^a^^,b^	BD, 324702
		NSG Males (10 weeks old)^a^	Jackson Laboratories, 005557
	TV	Heat Lamp^a^^,b^	–
		Mouse Restrainer^b^	–
*In vivo* monitoring		VetOne Fluriso^a^^,b^	MWI, 502017
		Luciferin Potassium Salt^b^	Regis Technologies Inc., 1-360222-200
		IVIS Spectrum In Vivo Imaging System^b^	Perkin Elmer
		Living Image Software^b^	Perkin Elmer
Tissue collection	Blood	Microtainer Tubes with K2EDTA	BD, 365974
		Single Edge Industrial Razor Blades^a^^,b^	VWR, 55411-050
		Eppendorf Tubes 5.0 ml	Eppendorf, 0030119487
	BM	1.5 ml Eppendorf Safe-Lock Tubes	Eppendorf, 022363328
		0.5 ml PCR Tube with Attached Cap, PP	Fisher Scientific, 14230200
		Spectrafuge 24-D Centrifuge	Labnet International, Inc.
		Dissection Tools^a^^,b^	–
	Lung	DPBS 1X w/out Ca, w/out Mg	ATCC, 30-2200
Tissue processing	Blood/BM	ACK Lysing Buffer^a^	Quality Biological, 118-156-101
	Lung	Collagenase/Hyaluronidase	Stemcell Technologies, 07912
		DNase I Stemcell Technologies, 07470)	Stemcell Technologies, 07470
		70 µm Nylon Cell Strainer	Falcon, 352350
		5 ml Syringe Luer-Lok Tip	BD, 309646
		14 ml PP Round-Bottom Tube	Falcon, 352059
		Petri Dish, Sterile, PS	Fisher Scientific, FB0875712
		I2400 Incubator Shaker^a^	New Brunswick Scientific
Flow cytometry		5 ml PS Round-Bottom Tube w. strainer cap	Falcon, 352235
		Vybrant DyeCycle Violet stain	Invitrogen, V35003
		Attune NxT Acoustic Focusing Cytometer	Thermo Fisher Scientific
		Attune NxT Focusing Fluid 1X^b^	Invitrogen, A24904
		Attune NxT Wash Solution^b^	Invitrogen, A24974
		Attune NxT 1X Shutdown Solution^b^	Invitrogen, A24975
		Attune NxT Performance Tracking Beads^b^	Invitrogen, 4449754
Analysis		FlowJo Analysis Software^b^	BD

a Use in multiple steps of protocol.

b Items of standard laboratory use not specifically mentioned elsewhere in this manuscript.

**Table 2.** Common trouble-shooting tips

Observation	Potential problem	Solution
Lung-specific BLI signal is still strongly present 3 days following tail vein injection.	Delayed clearing-of non-extravasated cells. May lead to reporting of non-extravasated CTCs as DTCs.	Wait and reperform BLI imaging daily until expected clearance is observed.
Collected less than 500 µl of blood	Limitations in blood collection technique can limit probability of finding rare CTCs.	Discovered CTCs can be reported as number of CTCs discovered over number of total cells analyzed.
Excess muscle tissue clogs Eppendorf during bone marrow centrifugation.	Can limit probability of finding rare DTCs.	Carefully clear away tissue blockage and re-centrifuge bone marrow
Bulk of sinewy/fatty material present in lysed bone marrow	This is to be expected.	Carefully remove insoluble material with a pipette tip.
GFP+ events present in tissue of mock-injected animals	Cross-contamination between samples at the level of reusable dissection tools, plastic consumables such as pipet tips, sample splashing, etc.	Use careful bench-top technique to prevent cross-contamination. Prepare spiked-in cell culture control samples first, before murine samples are collected. Set and/or cross-reference GFP Negative exclusion gate with mock injected tissues.
Obvious time-dependent shift in Vybrant Dye Cycle Violet intensity	Time-dependent overdevelopment of stain intensity.	Work in smaller batches of samples to limit large amount of time between end of stain incubation and data collection per sample.

The table provides troubleshooting tips for common problems likely to be encountered.

### Cell culture

#### PC3-GFP-Luc

Experiments were performed with the PC3-GFP-Luc prostate cancer cell line. The cell line was generated from standard PC3 cells (ATCC, CRL-1435) + pLentilox-EV-Luc luciferase expression vector (University of Michigan Vector Core), + pLenti-CMV-GFP-Neo (657-2) GFP expression vector (Addgene, Plasmid 17447). All cells were cultured with RPMI 1640 media, supplemented with 10% Fetal Bovine Serum and 1% Penicillin–Streptomycin antibiotic at 37°C and in 5% CO_2_. Cells were routinely lifted using TryplE following a single PBS wash. Cells were STR-profile authenticated and tested for *mycoplasma* contamination biannually (Genetica).

#### High-ploidy PC3-GFP-Luc

Where indicated, high-ploidy PC3-GFP-Luc cells were generated as previously described [6, 7]. Briefly, PC3-GFP-Luc cells were treated with 12 µM of cisplatin (resuspended in sterile PBS supplemented with 140 mM NaCl, according to manufacturer’s protocol) for 72 h. After 72 h, treatment was removed and replaced with complete media. Cells were injected 10 days after treatment removal.

### *In vivo* injection and monitoring

Animal studies were reviewed and approved by the Johns Hopkins University Animal Care and Use Committee.

#### Subcutaneous tumor injection

A suspension of 200,000 PC3-GFP-Luc cells resuspended in complete media (or a mock suspension of media alone) was injected into the right flanks of 10-week-old, male, NOD Scid Gamma (NSG) mice in a total volume of 100 µl (1:1 volume: volume with Matrigel). Subcutaneous injection of PC3-GFP-Luc cells created a palpable tumor within 2–3 weeks. After 6 weeks, it was possible to find viable CTCs in murine blood and DTCs in murine hind-limb bone marrow. Bioluminescent imaging (BLI) was used to ensure appropriate model progression: Mice were imaged weekly to track primary tumor growth kinetics.

#### Tail vein injection

A suspension of 100,000 PC3-GFP-Luc cells suspended in PBS (or a mock suspension of PBS alone) was injected into the lateral tail vein of 10-week-old, male, NSG mice in a total volume of 100 µl. Tail vein injection of PC3-GFP-Luc cells caused the cells to filter directly into pulmonary capillaries. After 3 days, it was possible to find viable DTCs in murine lung tissue. Tail vein-injected mice were imaged within 30 min of injection to confirm correct injection technique, indicated via the presence of strong lung signal and lack of tail-specific signal. Mice were imaged again immediately prior to tissue collection (3 days after injection) to confirm attrition of cells that stalled in lung capillaries but failed to extravasate into lung tissue, indicated by a large decline in lung signal. These kinetics have been previously reported: extravasation typically takes place within the first 24 h following initial arrest, and most non-extravasated cells are cleared out of capillary systems within the first 72 h following initial arrest [8].

### Tissue collection

Tissue Collection through Flow Cytometry sections must be performed on the same day.

#### Blood collection

Blood was collected 6 weeks following subcutaneous injection. Blood was collected via tail bleed from live mice. Mice were exposed to a heat lamp containing a 60 W lightbulb for 5 min to induce vasodilation. The tail was sliced with a clean razorblade over the caudal artery, and blood was collected dropwise directly into K2EDTA-treated microtainers. We aimed for as much blood as possible, averaging 500 µl per mouse. Mice were immediately completely anesthetized with isoflurane and euthanized via cervical dislocation and prepared for bone marrow collection. Following blood collection, K2EDTA tubes were immediately inverted 4 times to prevent blood clotting and stored on ice until ready to process.

#### Bone marrow collection

Bone marrow was collected 6 weeks following subcutaneous injection. Bone marrow was collected from the hind-limb bones of freshly euthanized mice using a standard centrifugation protocol [9]. Specifically, after live-animal blood collection, mice were completely anesthetized with isoflurane and euthanized via cervical dislocation. The right and left femurs and tibias of each mouse were dissected. The distal femoral epiphysial plate from each femur and the proximal tibial epiphysial plate from each tibia were removed to ensure access to red bone marrow. Bones were placed marrow-exposed side down in a 0.5 ml tube punctured with a small hole which was then nested into a 1.5 ml tube. Tubes were centrifuged at maximum speed for 30 s to collect bone marrow into the 1.5 ml tube, which was stored on ice until ready to process.

#### Lung collection

Lung tissue was collected 3 days following tail vein injection. Mice were euthanized via cervical dislocation following complete anesthetization with isoflurane. All five lobes of the lungs were removed through a chest cavity incision. All lobes were transferred to a fresh 50 ml conical tube containing enough Dulbecco’s PBS +2% Fetal Bovine Serum to cover the sample, which was stored on ice until ready to process.

### Tissue processing

#### Blood processing

Blood processing is best performed in batches of six to eight mice, staggering if more than six to eight mice is necessary. Samples classified as “Fixed” in Fig. 4 (Tissue Preparation Optimization) were fixed via a 10-m incubation in sufficient volume of 4% paraformaldehyde, followed by three 5-min washes with PBS. All wash centrifugation steps were performed at 1500*g* for 5 min at 4°C. Note that fixation was omitted for samples classified as “Live” in Fig. 4. Subsequently, samples classified as “Permeabilized” in Fig. 4 were subjected to permeabilization with 1% Saponin in PBS for 10 min. Permeabilization was omitted for samples classified as “Unpermeabilized” in Fig. 4. Following this, all samples classified as “Lysed” in Fig. 4 were individually transferred to 5 ml Eppendorf tubes and supplemented with ACK lysis buffer at a 1:4 blood: lysis buffer ratio. This solution was incubated on an end-over-end turner for 10 min. Lysis was omitted for samples classified as “Unlysed” in Fig. 4. Following incubation, all samples were centrifuged at 1500*g* for 10 min at 4°C. The supernatant was aspirated off, and the pellet was resuspended in 1 ml complete RPMI and stored on ice.

#### Bone marrow processing

Bone marrow processing is best performed in batches of six to eight mice, staggering if more than—six to eight mice is necessary. Samples classified as “Fixed” in Fig. 4 (Tissue Preparation Optimization) were fixed via a 10-min incubation in sufficient volume of 4% paraformaldehyde, followed by three 5-min washes with PBS. All wash centrifugation steps were performed at 1500  *g* for 5 min at 4°C. Note that fixation was omitted for samples classified as “Live” in Fig. 4. Subsequently, samples classified as “Permeabilized” in Fig. 4 were subjected to permeabilization with 1% Saponin in PBS for 10 min. Permeabilization was omitted for samples classified as “Unpermeabilized” in Fig. 4. Following this, all samples were resuspended in 200 µl PBS. Then, samples classified as “Lysed” in Fig. 4 were supplemented with 800 µl of ACK lysis buffer. This solution was incubated on an end-over-end turner for 10 min. Lysis was omitted for samples classified as “Unlysed” in Fig. 4. Following incubation, all samples were centrifuged at 1500 *g* for 10 min at 4°C. The supernatant was aspirated off, and the pellet was resuspended in 3 ml complete RPMI and stored on ice.

#### Lung processing

Lung processing is best performed in batches of six to eight mice, staggering if more than six to eight mice is necessary. Each set of lungs was transferred to a petri dish and minced into small pieces with a fresh straight-edged razor blade. Each sample was transferred to a 14 ml round-bottom tube and suspended in 5 ml of a solution containing 250 µl Collagenase/Hyaluronidase, 375 µl DNase I solution at 1 mg/ml, and 1.875 ml complete RPMI 1640 media. The samples were incubated at 37°C for 20 min with shaking, before being pushed through a fresh 70-micron strainer placed over a 50 ml conical tube using the rubber end of a fresh 5 ml syringe plunger. An aliquot of 45 ml of complete RPMI was used per sample to facilitate straining. Following straining, samples were centrifuged at 300 *g* for 10 min at room temperature, using a slow deceleration setting. Pellets were resuspended in 3 ml of ACK lysis buffer and incubated at room temperature for 3 min before adding 47 ml of complete RPMI. Samples were then centrifuged at 300 *g* for 10 min at room temperature, using a slow deceleration setting. Pellets were resuspended in 2 ml of complete RPMI and stored on ice.

### Flow cytometry

#### Staining samples

This protocol is best performed in batches of six to eight mice, staggering if more than six to eight mice is necessary. Samples were stained with Vybrant DyeCycle Violet following the manufacturer’s protocol of 1 µl dye per 1 ml cell suspension, assuming a maximum suspension concentration of 1,000,000 cells/ml. To avoid time-consuming, per-sample cell counting, we consistently used the following amounts: Blood: 1 µl/ml, for a total of 1 ml. (Note that on average, our blood collection approach yields 63,000 cells per ml). Bone Marrow: 5 µl/ml, for a total of 15 ml. (On average, our bone marrow collection approach yields 580,000 cells/ml). Lung: 5 µl/ml, for a total of 10 ml. (On average, our lung tissue collection approach yields 586,000 cells/ml). Control cells: 1 µl/1 ml, with a maximum concentration of 1,000,000 cells/ml. Stained samples were incubated for 30 min at 37°C protected from light and then stored on ice. Alternatively, for samples labeled “Fixed” in Fig. 4 (Tissue Preparation Optimization), an appropriate concentration of FxCycle Violet (following manufacturer’s protocol) was used in place of Vybrant DyeCycle Violet.

#### Running samples

Samples were run on Thermo-Fisher’s Attune NxT Acoustic Focusing Cytometer outfitted with a Blue/Red/Yellow/Violet four-laser, 16-channel configuration. The following four modifications were implemented to allow for analysis of a broader range of cell sizes: (i) the largest commercially available blocker bar was installed over the lasers; (ii) an alternative optical configuration was used (Supplementary Fig. S1); (iii) thresholding was performed using SSC rather than the standard FSC; and (iv) area scaling factors of 0.6 were used for all lasers, rather than the standard area scaling factors. With these modifications, data were collected in the SSC, VL1-A, VL2-A, and BL1-A channels. See Table 3 for the dichroic mirror and emission filters used per channel. Specifically, 500 µl of cell suspension was run for control samples at a flow rate of 200 µl/min. The entire volume of blood, bone, and lung tissue cell suspension was run at 1000 µl/min.

**Table 3.** Relevant channel specifications

Excitation laser	Channel	Dichroic mirror (nm)	Emission filter (nm)
Blue (488 nm, 50 mW)	SSC	Blank	488/10
Violet (405 nm, 50 mW)	VL1	Blank	405/10
Violet (405 nm, 50 mW)	VL2	415 DLP	440/50
Blue (488 nm, 50 mW)	BL1	495 DLP	530/30

Relevant channel specifications detailing the excitation laser, dichroic mirror, and emission filters used in the SSC, VL1, VL2, and BL1 channels.

### Necessary control samples

Inclusion of the following control samples is necessary for properly controlled analysis: (i) PC3-GFP-Luc cells, Unstained. (ii) PC3-GFP-Luc cells, Stained with Vybrant DyeCycle Violet. (iii) Matched tissue (blood, bone marrow, and/or lung) from an uninjected mouse, Unstained. (iv) Matched tissue (blood, bone marrow, and/or lung) from an uninjected mouse, Stained with Vybrant DyeCycle Violet. (v) Matched tissue (blood, bone marrow, and/or lung) from an uninjected mouse, spiked with PC3-GFP-Luc cells, Unstained. (vi) Matched tissue (blood, bone marrow, and/or lung) from an uninjected mouse, spiked with PC3-GFP-Luc cells, Stained with Vybrant DyeCycle Violet. (vii) When appropriate, PC3-GFP-Luc high-ploidy, Unstained. (viii) When appropriate, PC3-GFP-Luc high-ploidy, Stained with Vybrant DyeCycle Violet. (ix) When appropriate, Matched tissue (blood, bone marrow, and/or lung) from an uninjected mouse, spiked with PC3-GFP-Luc high-ploidy cells, Unstained. (x) When appropriate, Matched tissue (blood, bone marrow, and/or lung) from an uninjected mouse, spiked with PC3-GFP-Luc high-ploidy cells, Stained with Vybrant DyeCycle Violet.

### Data analysis

**Figure 2.**
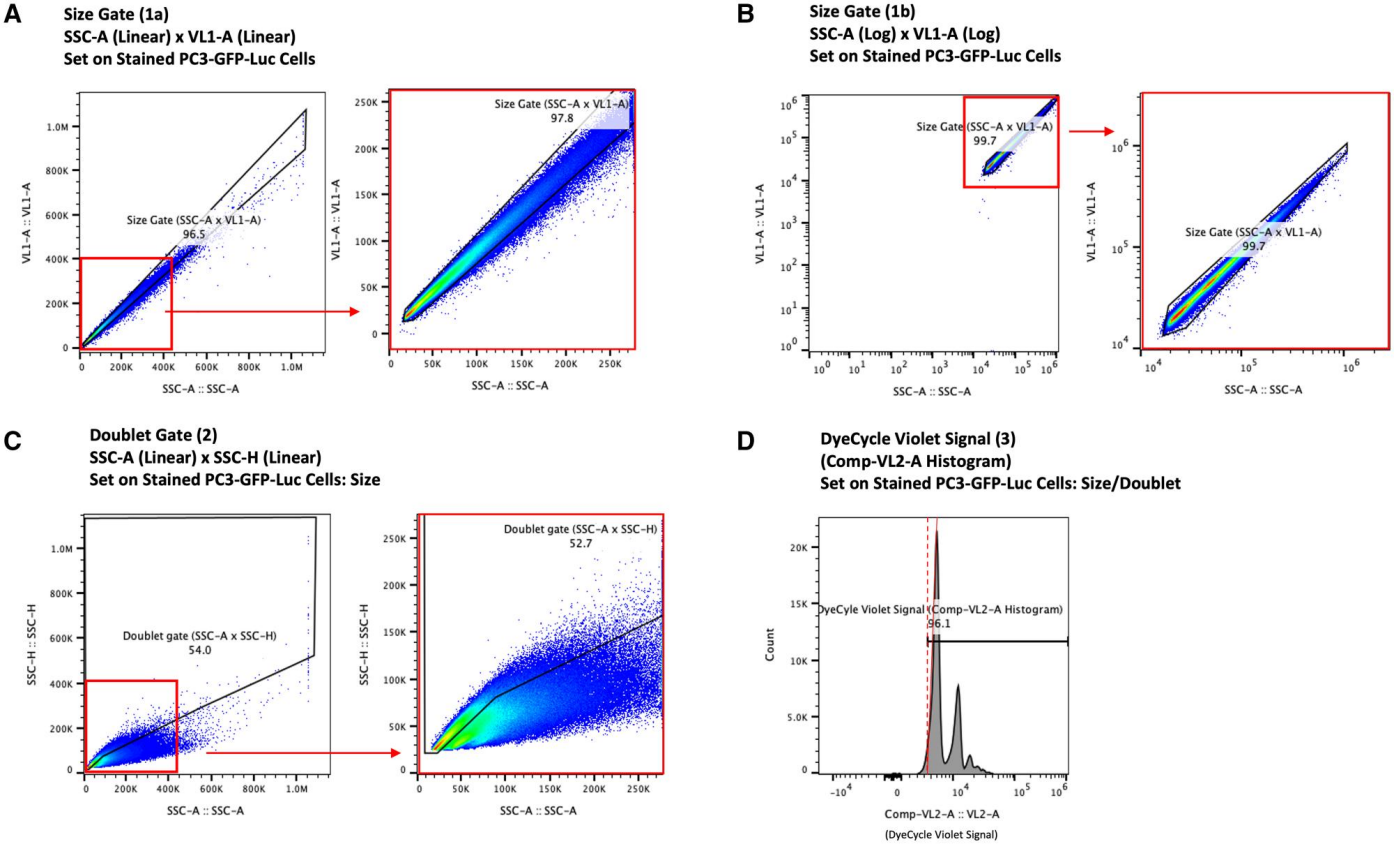
Pan tissue gating (Gates 1–3): (A) Size-exclusion Gate 1a defined using the distribution of control PC3-GFP-Luc cells stained with Vybrant DyeCycle Violet on an SSC-A linear versus VL1-A linear density plot. (B) Size-exclusion Gate 1b defined using the distribution of control PC3-GFP-Luc cells stained with Vybrant DyeCycle Violet on an SSC-A log versus VL1-A log density plot. (C) Doublet-exclusion Gate 2 defined by the distribution of control PC3-GFP-Luc cells stained with Vybrant DyeCycle Violet on an SSC-A linear versus SSC-H linear density plot. (D) Vybrant DyeCycle Violet unstained exclusion Gate 3 defined by the distribution of control PC3-GFP-Luc cells stained with Vybrant DyeCycle Violet on a Comp-VL2-A histogram plot.

**Figure 3.**
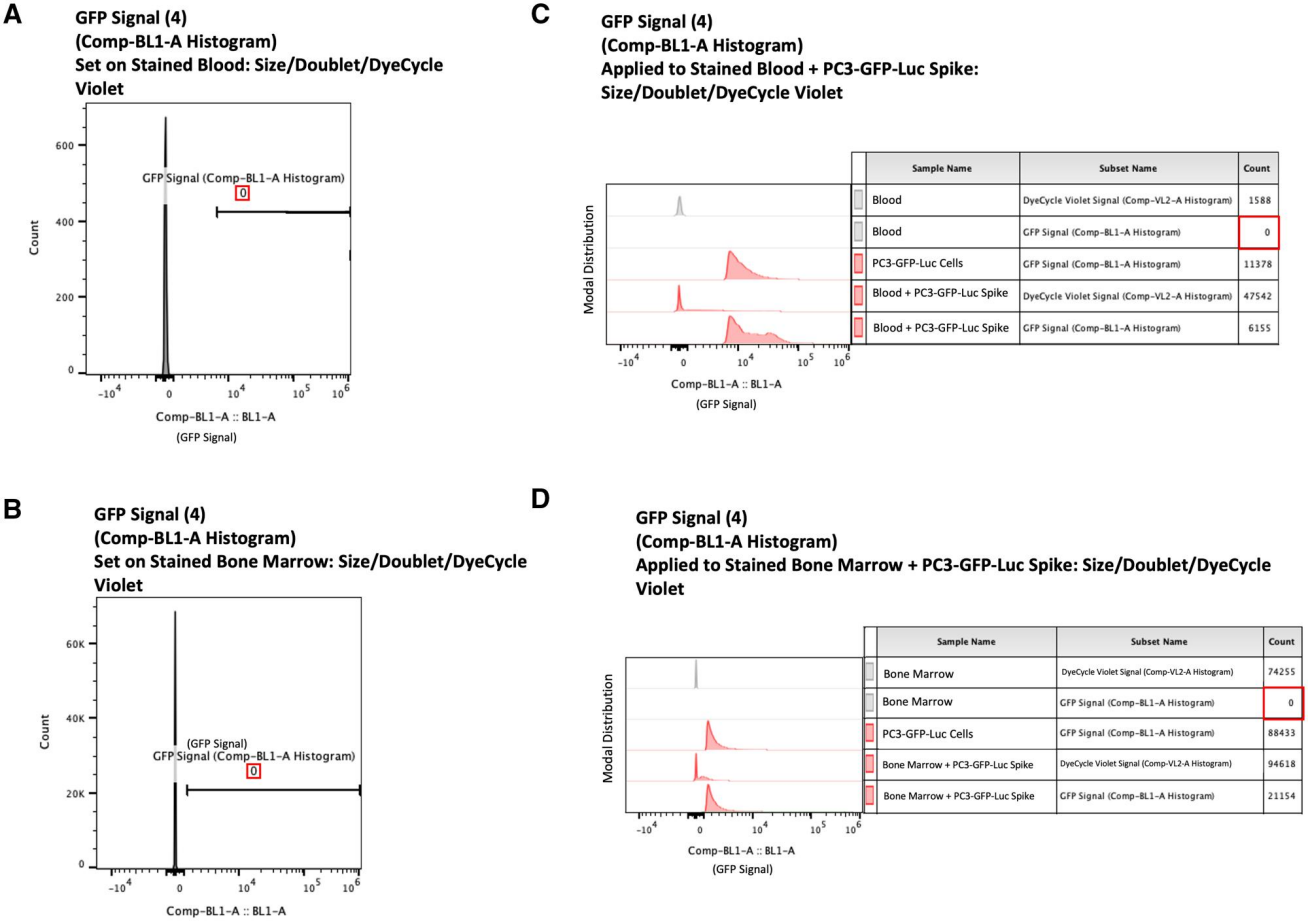
Tissue-specific gating (Gate 4): (A and C) Tissue-specific GFP-negative exclusion Gate 4 defined by the distribution of events analyzed in the uninjected control sample of murine blood stained with Vybrant DyeCycle Violet on a Comp-BL1-A histogram plot (A), and then applied to uninjected murine blood samples that have been spiked with PC3-GFP-Luc cells and then stained with Vybrant DyeCycle Violet (C). (B and D) Tissue-specific GFP-negative exclusion Gate 4 defined by the distribution of events analyzed in the uninjected control sample of murine bone marrow stained with Vybrant DyeCycle Violet on a Comp-BL1-A histogram plot (B), and then applied to uninjected murine bone marrow samples that have been spiked with PC3-GFP-Luc cells and then stained with Vybrant DyeCycle Violet (D).

FloJo analysis software was used to analyze raw data. Compensation was performed using the following control samples: (i) BLI Positive: Matched tissue (blood, bone marrow, and/or lung) from an uninjected mouse, spiked with PC3-GFP-Luc cells, Unstained. (ii) BL1 Negative: Matched tissue (blood, bone marrow, and/or lung) from an uninjected mouse, Unstained. (iii) VL2 Positive: Matched tissue (blood, bone marrow, and/or lung) from an uninjected mouse, Stained with Vybrant DyeCycle Violet. (iv) VL2 Negative: Matched tissue (blood, bone marrow, and/or lung) from an uninjected mouse, Unstained. The following gating strategy was used (Figs. 2 and 3): (i) Gate 1a: SSC-A linear axis density plot versus VL1-A linear axis density plot for size-based exclusion. (ii) Gate 1b: SSC-A logarithmic (log) axis density plot versus VL1-A logarithmic (log) axis density plot for size-based exclusion. (iii) Gate 2: SSC-A linear axis density plot versus SSC-H linear axis density plot for doublet exclusion. (iv) Gate 3: Comp-VL2-A histogram for Vybrant DyeCycle Violet unstained exclusion. (v) Gate 4: Comp-BL1-A histogram for GFP-negative exclusion.

## Results

We highlight the successful co-identification of blood-sourced CTCs and bone marrow-sourced DTCs from single mice using an optimized flow-cytometry approach. The identification of such rare events by flow cytometry requires stringent analysis. We developed and confirmed the following analysis pipeline. Note that prior to creation or application of any gates, compensation must be performed as outlined in Data Analysis section.

### Gating strategy

First, to eliminate contaminating small debris and large, irregularly shaped foreign matter, a size-exclusion gate (gate 1a) is defined. Gate 1a is set by the distribution of control PC3-GFP-Luc cells stained with Vybrant DyeCycle Violet on an SSC-A linear versus VL1-A linear density plot (Fig. 2A). Gate 1a is then either confirmed or minutely adjusted into size-exclusion gate 1B via the distribution of control PC3-GFP-Luc cells stained with Vybrant DyeCycle Violet on an SSC-A log versus VL1-A log density plot (Fig. 2B). Next, doublet-exclusion (gate 2) eliminates doublet pairs of cells that are clustered together. Gate 2 is set by the distribution of control PC3-GFP-Luc cells stained with Vybrant DyeCycle Violet on an SSC-A linear versus SSC-H linear density plot (Fig. 2C). To eliminate residual red blood cells that lack nuclear content, as well as dead or dying cells, an unstained-exclusion gate (gate 3) is defined to identify events that are not stained with DNA stain (Vybrant DyeCycle Violet). Gate 3 is set using control PC3-GFP-Luc cells stained with Vybrant DyeCycle Violet on a Comp-VL2-A histogram plot (Fig. 2D). Gates 1–3 are pan-tissue, as they are established using control cultured cells, and thus are appropriate to apply to flow analyte of any tissue type.

Each tissue has a unique level of background autofluorescence detected in the BL1 channel. Ergo, it is critical to set this gate individually per tissue type, to the lowest intensities that still exclude 100% of BL1 positive events. Gate 4 is a tissue-specific, GFP-negative-exclusion gate defined by the distribution of events analyzed in the uninjected control sample of the tissue of interest stained with Vybrant DyeCycle Violet on a Comp-BL1-A histogram plot (blood samples, Fig. 3A; bone marrow samples, Fig. 3B). This gate determines the tissue-specific, GFP-autofluorescence-threshold for reliable detection of CTCs and DTCs defined using GFP-positivity.

The previously defined pan-tissue gates 1–-3 are applied to spiked and stained blood and bone marrow samples that contain PC3-GFP-Luc cells added post-tissue processing in Supplementary Fig. S2A–D. The previously defined tissue-specific gate 4 is applied to spiked and stained blood and bone marrow samples that contain PC3-GFP-Luc cells added post-tissue processing in Fig. 3C and D, respectively.

### Optimal sample preparation for flow cytometric analysis

**Figure 4.**
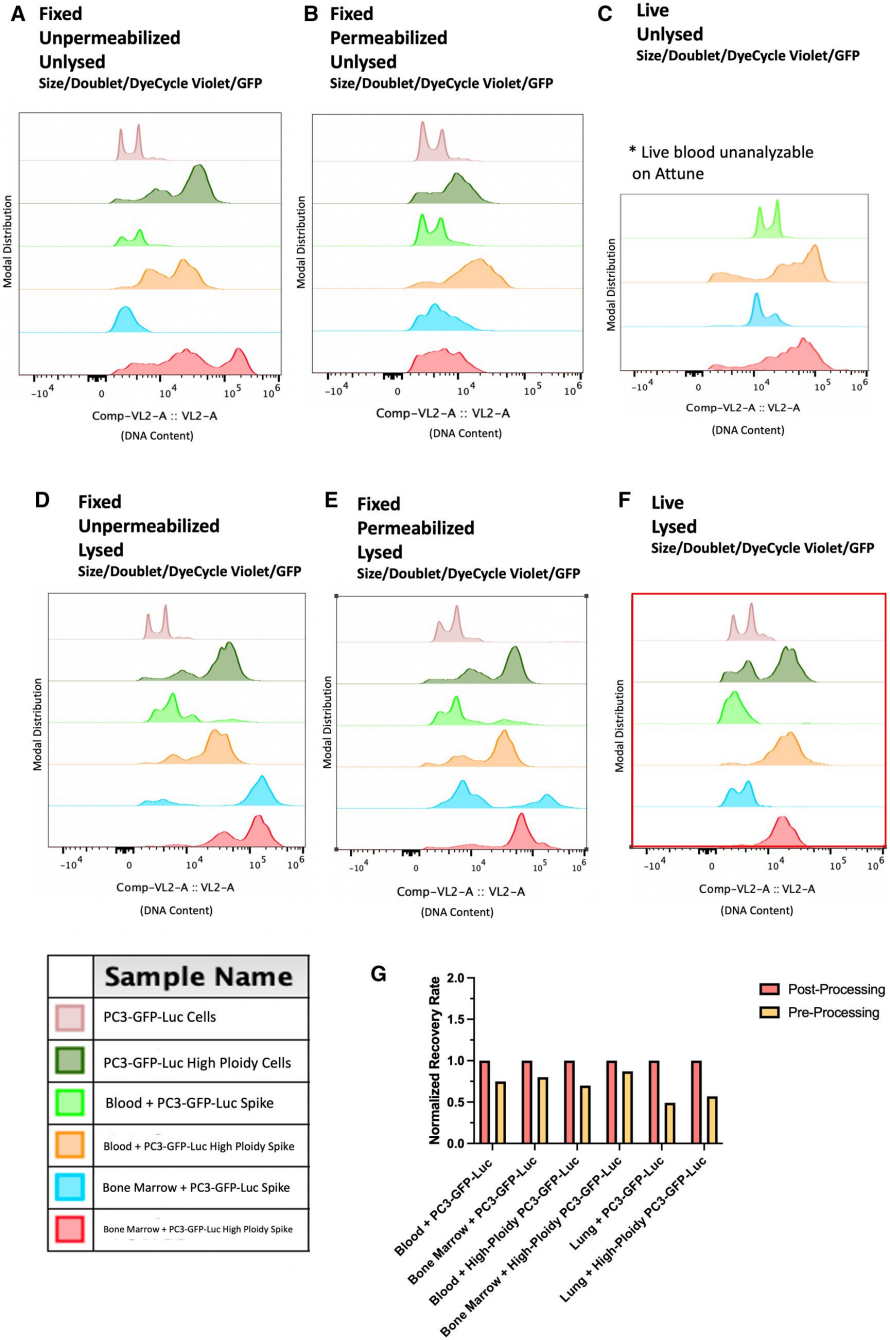
Tissue preparation optimization: (A–F) Comp-VL2-A histograms of PC3-GFP-Luc normal or high-ploidy cells alone, spiked into murine blood, or spiked into murine bone marrow, remaining following application of Gates 1–4. (A) Cells were fixed, but not permeabilized nor lysed preceding cytometry. (B) Cells were fixed and permeabilized, but not lysed preceding cytometry. (C) Cells were not fixed, not permeabilized, and not lysed preceding cytometry. (D) Cells were fixed, were not permeabilized, but were lysed preceding cytometry. (E) Cells were fixed, permeabilized, and lysed preceding cytometry. (F) Cells were not fixed nor permeabilized but were lysed preceding cytometry. (G) Comparison of the percent recovery rates of normal-ploidy and high-ploidy cells spiked into blood, bone marrow, and lung tissue prior to tissue processing versus following tissue processing.

We optimized the upstream tissue processing conditions for ideal cellular recovery. To do this, we spiked 1,000,000 PC3-GFP-Luc normal-ploidy (or large, high-ploidy cells, to ensure the finalized approach was appropriate for cells of various sizes) into blood or bone marrow harvested from adult, male, NSG mice and assayed various processing conditions. Our tested conditions included (i) fixed cells versus live cells, (ii) permeabilized cells versus unpermeabilized cells, and (iii) ACK lysed versus unlysed cells. We found that live, ACK lysed cells (both normal and high-ploidy) could be best recovered from both blood and bone marrow (Fig. 4F). Fixation with or without permeabilization and subsequent use of standard live/dead dyes commonly used to report DNA content resulted in both skewing and shifting of the reported DNA content of cells recovered from both blood and bone marrow tissue, as compared to the reported DNA content of controls cells in media (Figs. 4A–E). Additionally, live and unlysed blood samples were found to be unanalyzable on the Attune NxT Acoustic Focusing Cytometer due to frequent “bubble error” warnings (Fig. 4C). To test that the tissue processing steps do not selectively destroy GFP+ cells, we compared the percent recovery rate of GFP+ cells spiked into blood, bone marrow, or lung prior to tissue processing (pre-processing) versus following tissue processing (post-processing). We found that the processing steps only slightly diminish the percent recovery rate in blood and bone marrow, but reduce the recovery rate by nearly 50% in the lung (Fig. 4G).

### Sensitivity of flow cytometric approach for detection of rare cells in blood and bone marrow

**Figure 5.**
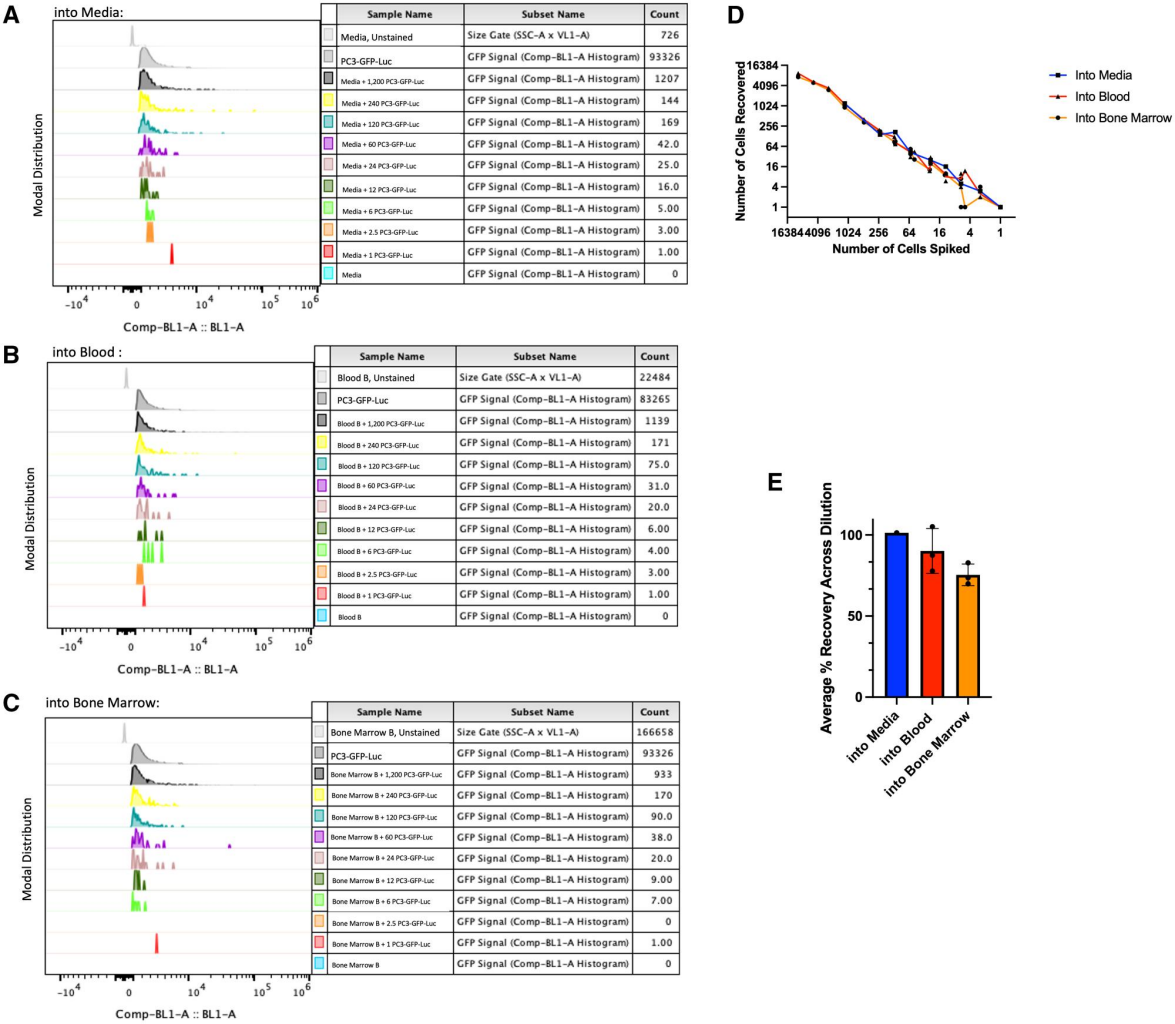
Limiting dilution: (A) Comp-BL1-A histogram of recovered PC3-GFP-Luc cells spiked into media via limiting dilution. (B) Representative example of Comp-BL1-A Histogram of recovered PC3-GFP-Luc cells spiked into pooled murine blood samples. (C) Representative example of Comp-BL1-A histogram of recovered PC3-GFP-Luc cells spiked into pooled murine bone marrow samples. (D) Number of cells spiked versus number of cells recovered from media, blood, and bone marrow samples across three biological replicates. (E) Average % Recovery of spiked cells in media, blood, and bone marrow across three biological replicates.

To determine the sensitivity of this approach, we spiked a limiting serial dilution of PC3-GFP-Luc cells into media alone or into blood or bone marrow samples harvested from adult, male, NSG mice. We measured the resulting detection (recovery) rates from three independent experiments (Fig. 5A–D). On average, across the dilution, 100% of cells spiked into media were recovered, while cells spiked into blood and bone marrow were recovered at rates of 90.13% and 75.41%, respectively (Fig. 5E). Even at very low inputs, spiked-in cells could repeatedly be detected (Fig. 5).

### CTCs and DTCs detected in tissues collected from tumor-bearing mice

**Figure 6.**
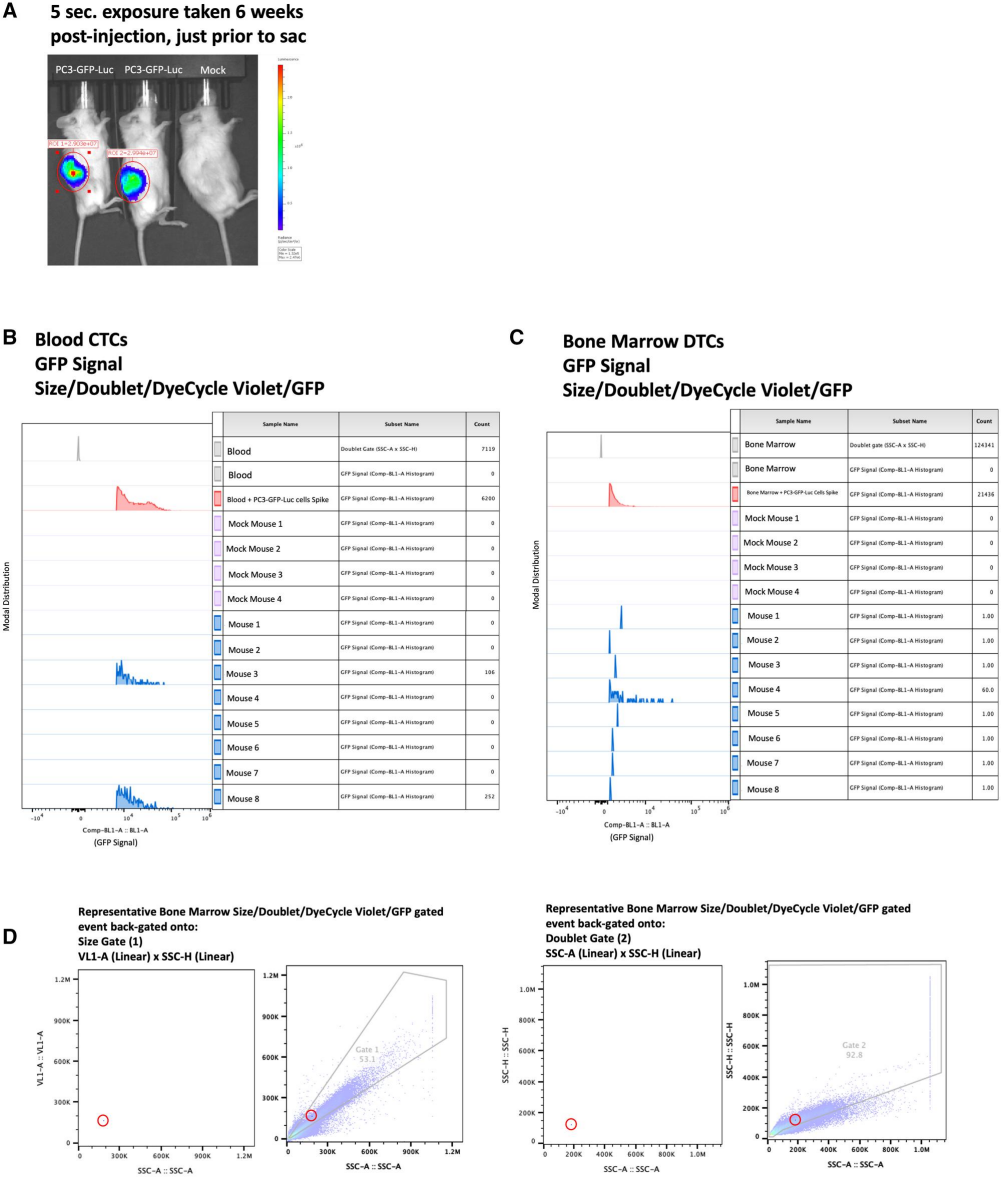
CTCs and DTCs Recovered from Blood and Bone Marrow Following Subcutaneous Injection: (A) Representative BLI image of mice 6 weeks after subcutaneous injection of PC3-GFP-Luc cells or mock. (B) Comp-BL1-A histogram of blood-recovered CTCs from a subcutaneous metastasis model following application of Gates 1–4. (C). Comp-BL1-A histogram of bone-marrow recovered DTCs from a subcutaneous metastasis model following application of Gates 1–4. (D) Representative example of recovered GFP+ metastatic events back-gated onto Gates 1 and 2, demonstrating they are do not qualify as “edge-events.”

In the example data showcased in Fig. 6, blood CTCs and bone marrow DTCs were recovered from mice 6 weeks after subcutaneous tumor injection (Fig. 6A). Examples of CTCs identified in the blood of injected mice are included in Fig. 6B: 106 and 252 CTCs were identified per mouse. Examples of DTCs identified in the bone marrow of injected mice are included in Fig. 6C. Most frequently, 1 DTC was identified per mouse. No CTCs or DTCs were identified in mock-injected mice (Fig. 6B and C). The total numbers of cells analyzed per mouse are available in Table 4 and Supplementary Fig. S5E. Percent GFP+ cells recovered normalized to total number of cells analyzed per mouse are available in Supplementary Fig. S5F.

**Table 4.** Total number of cells analyzed per mouse.

Blood sample	No. of blood cells analyzed
Mock 1	112,966
Mock 2	28,916
Mock 3	87,351
Mock 4	63,587
Mouse 1	19,658
Mouse 2	50,178
Mouse 3	98,655
Mouse 4	129,999
Mouse 5	14,446
Mouse 6	104,011
Mouse 7	28,185
Mouse 8	18,720
**Average**	**63,056**

**Lung sample**	**No. of lung cells analyzed**

Mock 1	2,134,048
Mock 2	573,527
Mock 3	302,801
Mock 4	1,260,443
Mouse 1	1,028,106
Mouse 2	1,661,579
Mouse 3	1,255,235
Mouse 4	1,671,525
HP Mouse 1	886,458
HP Mouse 2	1,071,510
HP Mouse 3	1,458,529
HP Mouse 4	767,780
**Average**	**1,172,628**

**Bone marrow sample**	**No. of bone marrow cells analyzed**

Mock 1	2,558,703
Mock 2	869,585
Mock 3	2,545,314
Mock 4	2,545,314
Mouse 1	2,210,123
Mouse 2	1,584,095
Mouse 3	1,494,351
Mouse 4	1,341,169
Mouse 5	743,528
Mouse 6	2,501,825
Mouse 7	1,066,351
Mouse 8	2,328,598
**Average**	**1,740,801**

The table shows the total number of cells analyzed per mouse as reported in blood and bone marrow (Fig. 6) and in lung (Supplementary Fig. S5). HP = High-Ploidy

We also tested the applicability and adaptability of this protocol to other tumor models. First, we confirmed that this approach is sufficient to detect large cancer cells in mouse blood and bone marrow. High-ploidy cells, which we have reported to be many-fold larger than PC3 cells [6], were spiked into blood and bone marrow. Using the same gating approach, we modified the gates to be appropriate for the sample of interest. To ensure that gate 1 is appropriate for high-ploidy cells, the axes should be adjusted to include the northwest corner of the density plot (Supplementary Fig. S4A for blood samples and Supplementary Fig. S4B for bone marrow samples). To make certain that gate 2 is appropriate for high-ploidy cells, the axes should be adjusted so inclusion of the western edge of the density plot is ensured (Supplementary Fig. S4C). Various tissue-specific gate 4s are applied to high-ploidy cell control samples and high-ploidy cells spiked into blood and bone marrow tissue in Supplementary Fig. S4E.

To demonstrate this system is amenable to recovery of rare cancer cells from solid organ types, we evaluated DTCs detected from lungs of animals subjected to tail vein injection of either normal ploidy PC3-GFP-Luc cells or high-ploidy PC3-GFP-Luc cells (Supplementary Fig. S5A). Lungs were collected 3 days following injection (Supplementary Fig. S5B). The gating strategy was followed as described above (Supplementary Fig. S3) and was ensured to be appropriate for detection of both normal-ploidy (typical-sized) PC3-GFP-Luc cells as well as high-ploidy (large) PC3-GFP-Luc cells in lung tissue (Supplementary Fig. S4). We found that all mice had either one or two DTCs (Supplementary Fig. S5). Total numbers of cells analyzed per mouse are available in Table 4. Finally, we verified the ability of this approach to enable phenotyping of cells of interest by performing additional ploidy analysis on identified lung DTCs. The ploidy analysis showed that all DTCs recovered in mice injected with normal cancer cells displayed a typical genomic content, while all DTCs recovered in mice injected with high-ploidy cancer cells had increased genomic content consistent with their high-ploidy status (Supplementary Fig. S5D). This additional analysis was performed using detection of the Vybrant DyeCycle Violet stain in the VL2 channel and typifies the kind of supplementary analyses that can be complexed with this approach for further CTC and DTC characterization.

## Discussion

Rapid and reliable CTC and DTC detection forms a major underpinning of rigorous *in vivo* metastasis research. While many cancer cells initiate metastasis, very few can complete it. In other words, many motile or invasive cells never thrive as CTCs, and many CTCs never thrive as DTCs. As such, there is a need for creative, synergistic metastasis research approaches that provide a more detailed understanding of the specific failure points of various metastasis models along the metastatic cascade. Here, we present an innovative approach to rare cell detection that allows for the simultaneous comparison of multiple steps of the cascade within one model system through the co-identification of CTCs and DTCs from single animals. This experimental design is preferred because it eliminates cross-cascade inference-making. For example, observation of increased numbers of CTCs in the circulation cannot be used to independently predict future increased metastatic burden. Without orthogonal DTC data, the CTC data alone remain too limited to assess the presence (or absence) of a possible extravasation-incompetency bottleneck, for example.

Using this approach on the Attune NxT Acoustic Focusing Cytometer, we were able to detect abundant CTCs within 500 µl of blood from mice. We utilized an altered optical configuration inside of our flow cytometer that accommodated a broader range of cell sizes than the standard configuration. This allowed us to apply the methodology to normal-sized, normal-ploidy cancer cells, as well as larger-sized, high-ploidy cancer cells, which represent an emerging focal metastasis research area. This altered configuration introduces a “blank” dichroic mirror and 405/410 emission filter into the VL1 channel, which works alongside standard SSC parameters to map the distribution of cell size, shape, and surface complexity. The dichroic mirror and emission filter compatible with Vybrant DyeCycle Violet are shifted from the standard VL1 channel to the VL2 channel, and the standard VL2 dichroic mirror and emission filter are removed. The recommended standard GFP parameters are used in the BL1 channel. Interestingly, this modified configuration eliminates the otherwise standard necessity to lyse any red blood cells present in samples by avoiding the size, number, and autofluorescence-mediated “swarming effects” produced by cytometers with standard laser configurations. Accordingly, we tested the accuracy of the DNA-content readout of spiked-in GFP+ cells subsequently recovered from the blood and bone marrow of Lysed versus Unlysed samples. We found that the DNA content histogram of GFP+ cells recovered from Lysed samples (versus Unlysed) was most like that of control “non-spiked” GFP+ cells. So, while red blood cell lysis is not technically necessary here, we found it increases the accuracy of DNA content read-out.

The tissue preparation methods used here successfully produced single-cell suspensions of live cells in blood, bone marrow, and lung tissue samples that could be analyzed for GFP positivity and DNA content. While it is true that biological tissue autofluorescence will often mask as positive GFP signal due to inherent overlap in emission spectra, we are confident that the GFP signals observed in these experiments are properly controlled and gated for, and thus are not the result of autofluorescence. This notion is supported by (i) the lack of GFP+ events in samples of blood, bone marrow, and lungs taken from uninjected mice (Fig. 6 and Supplementary Fig. S5), (ii) the lack of GFP+ events in samples of blood, bone marrow, and lungs taken from mock-injected mice (Fig. 6 andSupplementary Fig. S5), and (iii) the appropriate scaling of GFP+ events in limiting dilution contexts (Fig. 5). Furthermore, we are confident that metastatic events identified here are not “edge” events produced through indiscriminate gating: when final metastatic events are back-gated onto the axes originally used to delineate size and doublet exclusion gates, the events are not near the gate barriers. In other words, they can be found squarely in the center of the included populations, and thus are not at risk of exclusion if any gates were to be minorly adjusted (Fig. 6D).

We assume the lack of detected CTCs in some tested mice is due to rare number or lack of CTCs in the blood at the time of blood collection, compounded by our inability to assay the entire blood volume of the animal. We have no evidence to suggest it was due to protocol or instrument failure. It is widely appreciated that CTCs are present at exceedingly low concentrations, necessitating analysis of a large number of blood cells. Here, as many blood cells as possible were analyzed, averaging 63,000 per animal.

We detected both bone marrow-derived and lung-derived DTCs in every animal analyzed, with an average number of 1,741,000 bone marrow cells and 1,173,000 lung cells analyzed per animal (Table 4). The limited number of DTCs identified in comparison to the number of CTCs identified aligns with clinical reports of an extravasation-bottleneck. In one animal, 60 bone DTCs were identified, indicating possible, if not likely, early colonization of a DTC into a micro-metastatic lesion as yet too small to be visualized via BLI prior to the experimental endpoint.

We show that our method is amenable to adaptation for evaluation of diverse cells of interest and tissues of origin. First, it is compatible with analysis of solid organs, such as lung tissue following tail vein injection. Additionally, it is compatible with a wide range of cell sizes and can be complexed with other fluorescent-based markers to further characterize recovered CTCs and DTCs. To demonstrate this, we successfully characterized the increased DNA content of cells known to be high-ploidy by adding a violet fluorescent DNA content dye and obtaining a confirmatory read-out of ploidy. Any other markers of interest could easily be included, assuming appropriate optical channel diversity. Of note, while the GFP signal in the BL1 channel was markedly increased at the per-cell level in high-ploidy cells compared to normal ploidy cells, this increased GFP signal intensity did not spill-over into any of the other 3 laser’s 11 detection channels. Increased GFP signal intensity in high-ploidy cells is consistently observable in tissue culture and is likely due to increased genome-wide protein expression (including of the GFP transgene) in these cells, which itself is potentially driven by the cells’ increased DNA content.

Some potential limitations of our system include the reliance on constitutive intracellular GFP expression. Though widely used in both immunocompromised and immunocompetent animal models, the immunoreactivity of GFP has been reported even in the immunocompromised setting [10]. Such immunoreactivity has the potential to limit the survival of GFP+ CTCs and DTCs in the animal over extended periods of time, artificially reducing the read-out of metastatic potential in our model systems. Additionally, it has been reported that GFP expression can deteriorate over time as GFP-tagged cells can be prone to die more rapidly than non-tagged cells [11]. Second, we developed and optimized this protocol with Thermo-Fisher’s Attune NxT Acoustic Focusing Cytometer. While most of the flow cytometer hardware and software settings that we used to achieve these results are temporary and easily interchangeable, the required installation of the largest commercially available blocker bar over our machine’s internal laser system is a permanent modification. We have not tested the reliability of this approach using a machine that does not contain the blocker bar, though we hypothesize that this approach would be suitable for analysis of normal-sized cancer cells on other flow cytometers equipped with a standard setup. A potential improvement to this protocol would be to optimize it for imaging-flow cytometry, to obtain visual information about detected CTCs and DTCs that could inform future experimentation. Common problems and appropriate troubleshooting solutions are included below in Table 2.

Our method is comparatively simple and non-laborious compared to existing CTC and DTC detection methodologies. Existing CTC and DTC detection protocols, including those developed in our laboratory, often rely on label-dependent detection via immunofluorescent imaging, magnetic particles, or microfluidics. These approaches are primarily optimized to delineate CTCs using expression of epithelial markers. Thus, they are limited by potential heterogeneous marker expression and frequent upregulation of epithelial-to-mesenchymal transition proteins. For example, some common epithelial markers such as CD326 have been found to be both present and absent in CTCs [12]. Additionally, such approaches are frequently limited to low sample volume and are hard to automate [13]. Other frequently cited CTC detection approaches include Prostate-Specific Membrane Antigen( PSMA)-specific cell surface labeling [14] or Alu element sequencing [15]. Though these methods have undoubtedly proved useful and accurate in the past, such time-intensive and laborious approaches are much better suited for downstream validation testing of any promising preliminary results yielded using our swift and simple approach.

Our previous murine CTC and DTC detection approach utilized an immunofluorescence-imaging-based detection technique. Though it was not dependent on epithelial or cancer-specific markers, it required labor-intensive smearing of harvested cells on slides, followed by mouse versus human cell-specific differential immunofluorescent staining and time-intensive slide scanning using a classifier detection system [16, 17]. The abundance of cells present in the bone marrow collected from two hind limbs (femur and tibia) of an average adult male mouse frequently required more than the single-run maximum limit (eight slides) of the Metafer detection system. Considering an eight-slide scan time of 12 h, it would take upwards of one month to scan a single experiment’s worth of slides. Furthermore, though the automated classifier-based detection system was effective in identifying potential human-origin CTC or DTCs among murine cells based on differential fluorescence, human verification was still required to remove about 30% of called events as false positives. Finally, this immunofluorescence-based approach required specialized microscopy platforms not readily available to all users. In recent years, several label-free detection methods have been developed, such as microfiltration, density gradient centrifugation, direct imaging, and dielectrophoresis. While free of the common constraints of label-based detection methods, these alternative approaches have been noted to yield low purity and large loss of cells [13]. Our new flow cytometry-based technique does not require post-tissue collection labeling, yet also is not limited by issues of purity. Though the GFP-expression dependency of this approach limits its translation to patients in the clinic, it successfully provides a new tool for metastasis researchers to obtain a rapid first-pass understanding of the metastatic potential of their cells of study under their conditions of interest. It is sufficiently high throughput for large tissue volumes, yet also comparatively rapid with a data collection time of only several hours. Combined, these features create possibility for investigators to easily pursue higher-powered comparative experiments with increased numbers of independent variables. This approach is also simple and accessible: Flow cytometry is a common technique that is used across multiple fields and is frequently available in laboratories and core facilities. Furthermore, we have demonstrated that this method is also reliable and widely adaptable for use by metastasis researchers. Finally, as aforementioned, this technique allows for the simultaneous detection and phenotyping of rare cells from multiple metastasis-relevant tissues per animal, thus increasing both the breadth and depth of collectable data.

## Supplementary Material

bpae026_Supplementary_Data

## Data Availability

Most data generated and analyzed during this study are included in this published article and its supplementary information files. Additional data are available from the corresponding author on reasonable request.
