# Supplementary figures and images for: Multiparameter flow cytometric detection and analysis of rare cells in in vivo models of cancer metastasis
Source: Biol Methods Protoc. 2024 Apr 27;9(1):bpae026. doi: 10.1093/biomethods/bpae026 (PMC11088742; doi:10.1093/biomethods/bpae026)

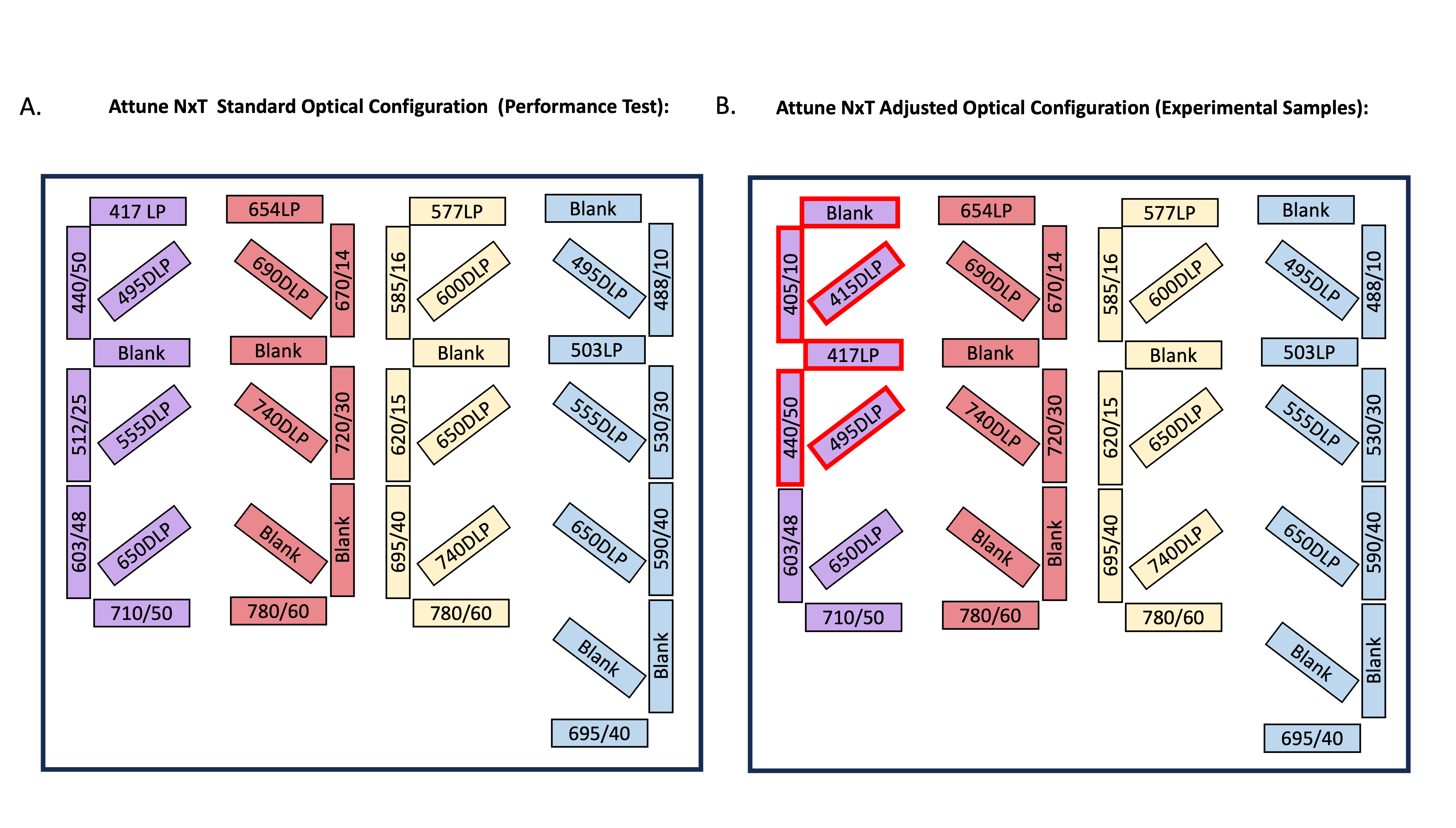

Supplement: bpae026_Supplementary_Data [file bpae026_supplementary_data.zip › Sup. Figure 1.png]

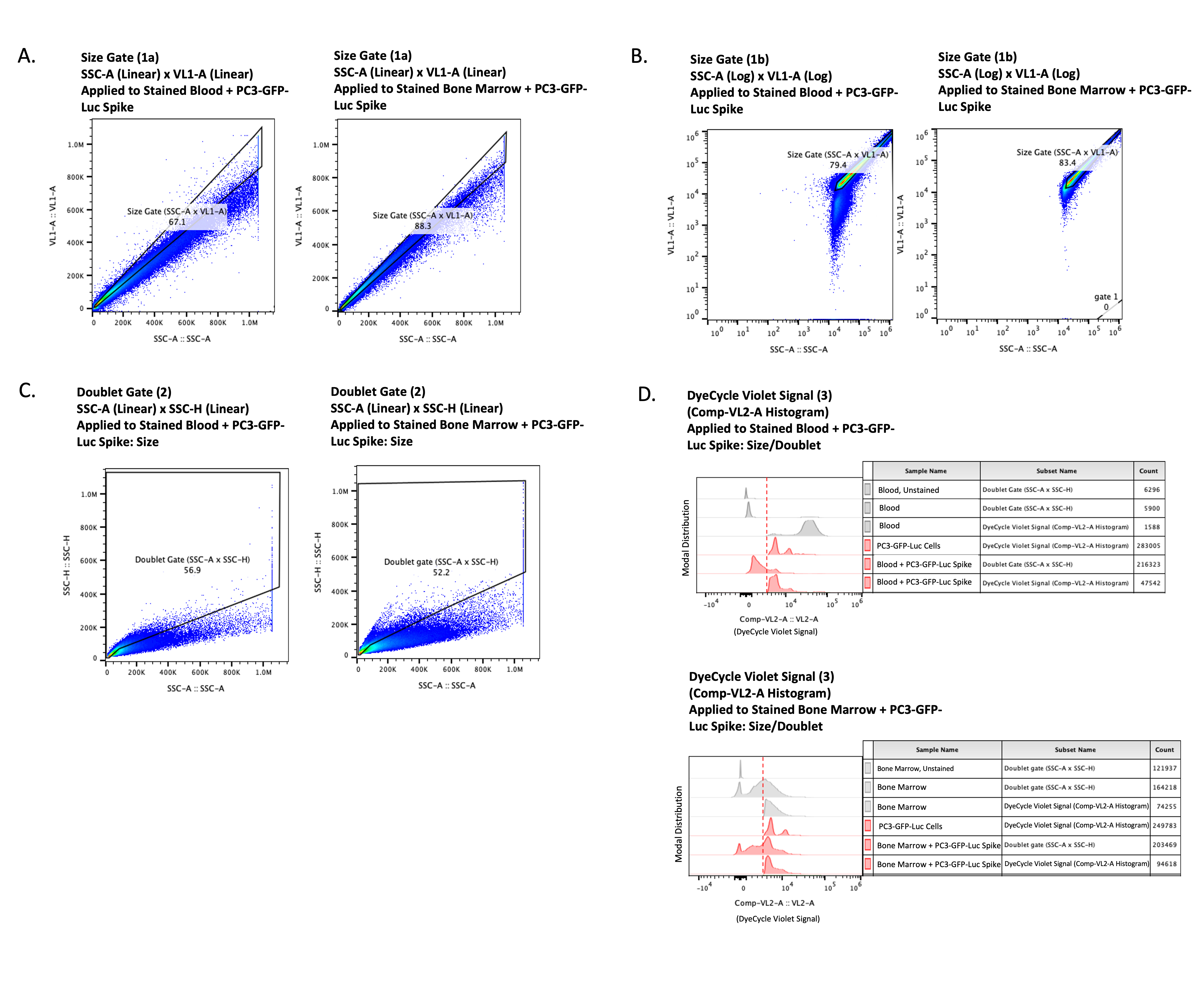

Supplement: bpae026_Supplementary_Data [file bpae026_supplementary_data.zip › Sup. Figure 2.png]

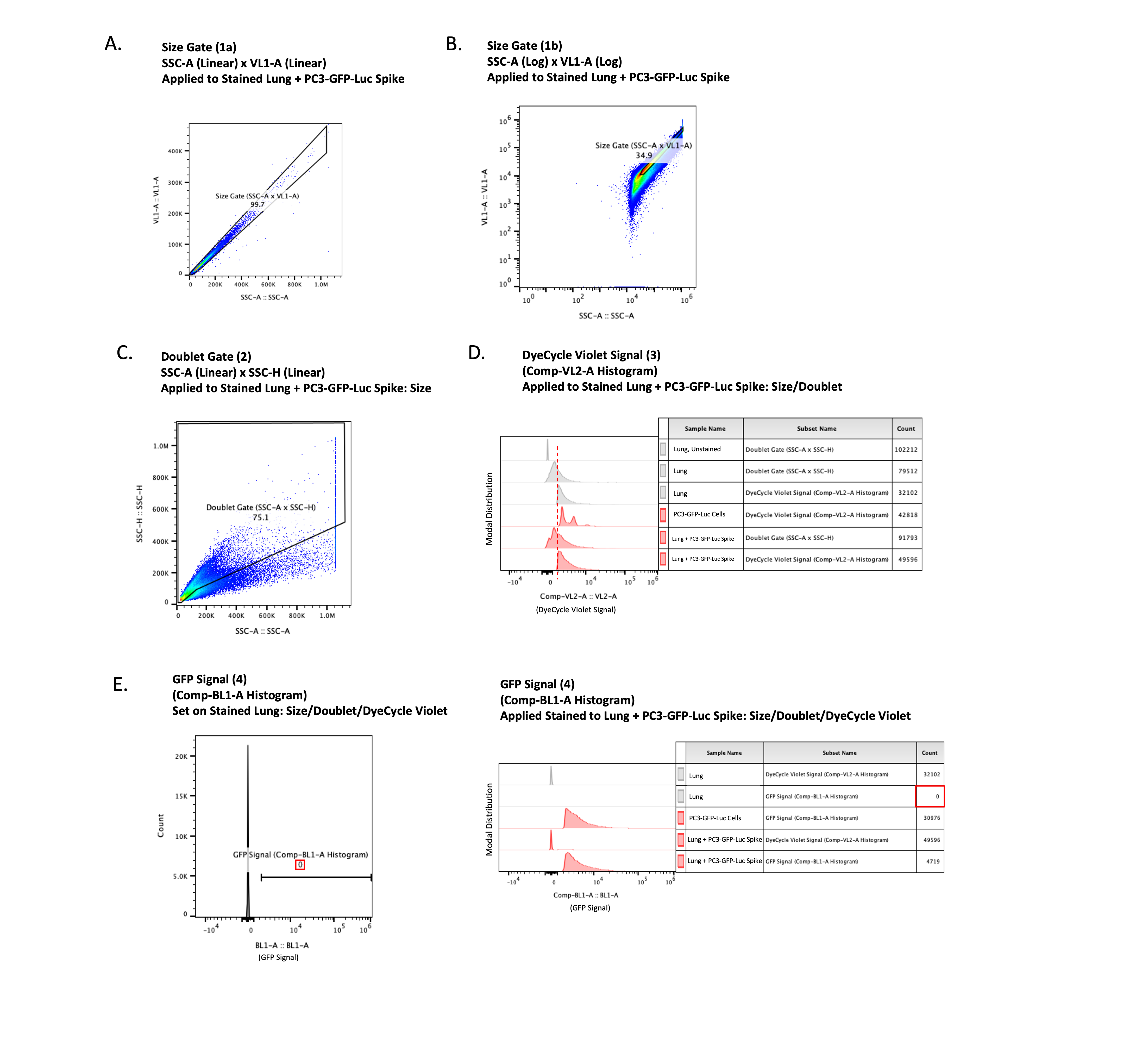

Supplement: bpae026_Supplementary_Data [file bpae026_supplementary_data.zip › Sup. Figure 3.png]

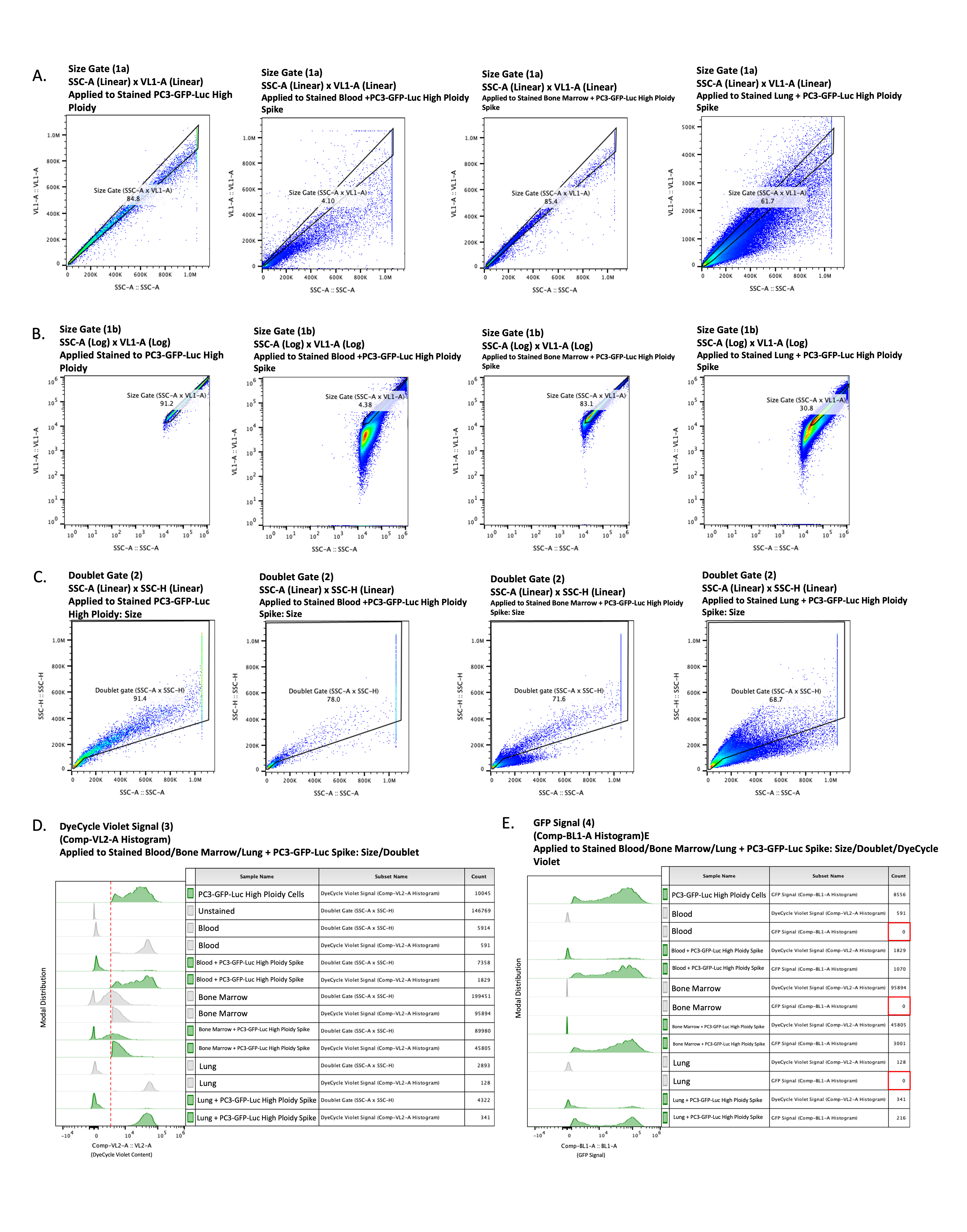

Supplement: bpae026_Supplementary_Data [file bpae026_supplementary_data.zip › Sup. Figure 4.png]

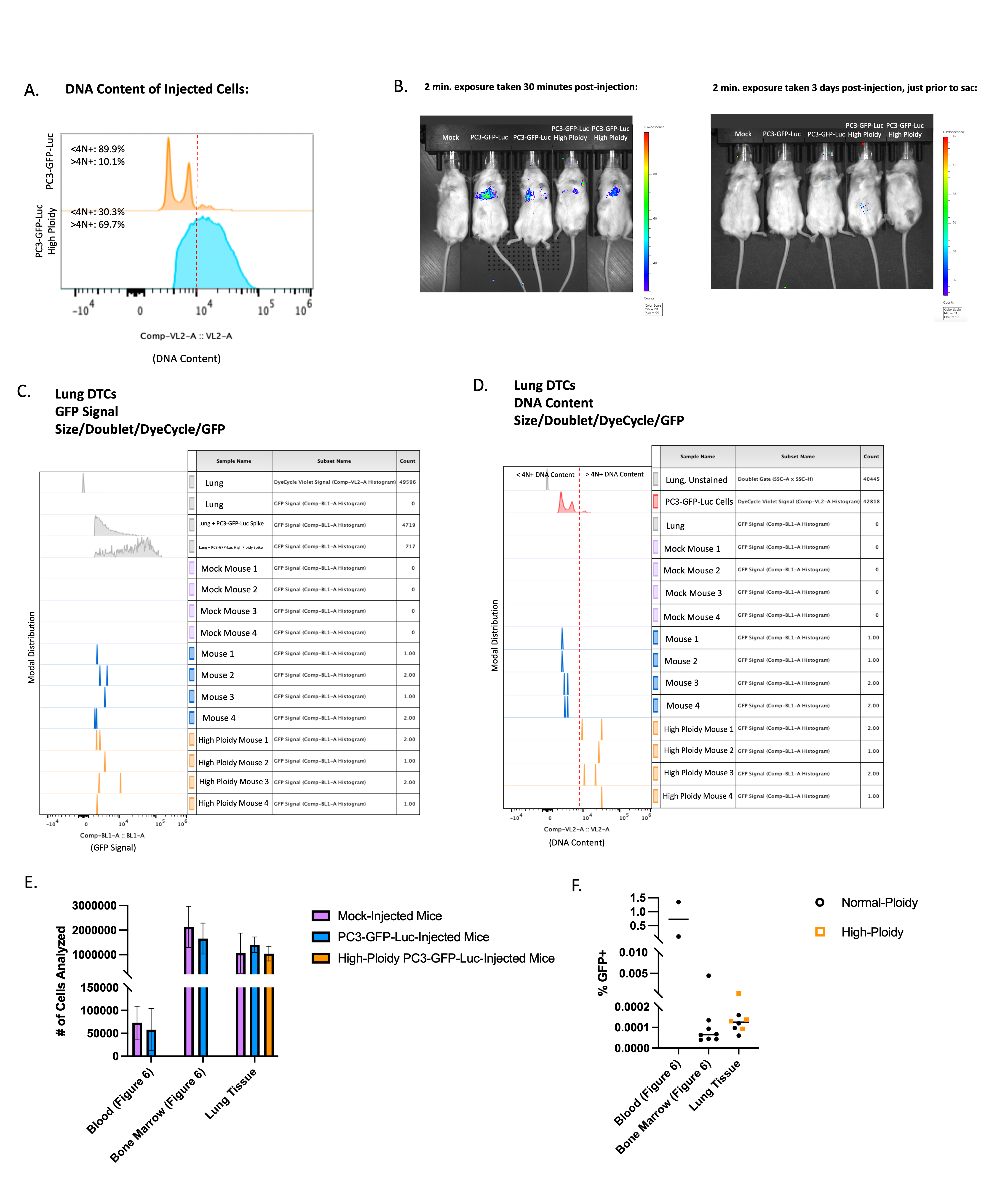

Supplement: bpae026_Supplementary_Data [file bpae026_supplementary_data.zip › Sup. Figure 5.png]
